# Variants of the Low Oxygen Sensors EGLN1 and HIF-1AN Associated with Acute Mountain Sickness

**DOI:** 10.3390/ijms151221777

**Published:** 2014-11-26

**Authors:** Enhao Zhang, Jihang Zhang, Jun Jin, Jun Qin, Huijie Li, Lan Huang

**Affiliations:** Institute of Cardiovascular Diseases of People’s Liberation Army, Department of Cardiology, Xinqiao Hospital, Third Military Medical University, Chongqing 400037, China; E-Mails: zhangeh@tmmu.edu.cn (E.Z.); sailingz@gmail.com (J.Z.); Jun_Jin@163.com (J.J.); Qinjun198@126.com (J.Q.); lihuijie001@163.com (H.L.)

**Keywords:** acute mountain sickness, Egl nine homolog 1 (EGLN1), hypoxia-inducible factor 1-α inhibitor (HIF-1AN), single nucleotide polymorphism, haplotype

## Abstract

Two low oxygen sensors, Egl nine homolog 1 (EGLN1) and hypoxia-inducible factor 1-α inhibitor (HIF-1AN), play pivotal roles in the regulation of HIF-1α, and high altitude adaption may be involved in the pathology of acute mountain sickness (AMS). Here, we aimed to analyze single nucleotide polymorphisms (SNPs) in the untranslated regions of the *EGLN1* and *HIF-1AN* genes and SNPs chosen from a genome-wide adaptation study of the Han Chinese population. To assess the association between *EGLN1* and *HIF-1AN* SNPs and AMS in a Han Chinese population, a case–control study was performed including 190 patients and 190 controls. In total, thirteen SNPs were genotyped using the MassARRAY^®^ MALDI-TOF system. Multiple genetic models were tested; The Akaike’s information criterion (AIC) and Bayesian information criterion (BIC) values indicated that the dominant model may serve as the best-fit model for rs12406290 and rs2153364 of significant difference. However, these data were not significant after Bonferroni correction. No significant association was noted between AMS and rs12757362, rs1339894, rs1361384, rs2009873, rs2739513 or rs2486729 before and after Bonferroni correction. Further haplotype analyses indicated the presence of two blocks in *EGLN1*; one block consists of rs12406290-rs2153364, located upstream of the *EGLN1* gene. Carriers of the “GG” haplotype of rs12406290-rs2153364 exhibited an increased risk of AMS after adjustments for age and smoking status. However, no significant association was observed among *HIF-1AN* 3'-untranslated region (3'-UTR) polymorphisms, haplotype and AMS. Our study indicates that variants in the *EGLN1* 5'-UTR influence the susceptibility to AMS in a Han Chinese population.

## 1. Introduction

Acute ascent to high-altitude environments can result in a medical condition referred to as acute mountain sickness (AMS) in accordance with Lake Louise score system (LLss) [1]. In China, millions of people travel to high altitudes for recreation, work and pilgrimage; therefore, many people are at risk of developing AMS. However, individuals, families and populations differ in their capacity to cope with the challenge [2]. AMS has become a general complaint that affects the ability to work at high altitudes. Unacclimatized people may even develop high-altitude cerebral edema (HACE), a lethal encephalopathic disease.

Although the pathology of AMS is not clear, hypobaric hypoxia serves as the primary etiology. Low oxygen concentrations inhibit hydroxylase activity and increase hypoxia-inducible factor-1α (HIF-1α) levels. HIF-1α accumulation and the subsequent up-regulation of vascular endothelial growth factor (VEGF) potentially contribute to basement membrane damage, and exaggerated edema might be involved in the pathophysiological process of AMS [3]. Two oxygen sensors, prolyl hydroxylase domain protein 2 (PHD2), also known as Egl nine homolog 1 (EGLN1), and factor inhibiting HIF-1α (FIH-1), also known as hypoxia-inducible factor 1-α inhibitor (HIF-1AN), play pivotal roles in HIF-1α pathway regulation.

EGLN1 is a 46-kDa enzyme that consists of two functional domain, *N*-terminal domain and *C*-terminal domain. *N*-terminal domain is homologous to MYND zinc finger whereas *C*-terminal domain is homologous to 2-oxoglutarate dioxygenases. EGLN1 hydroxylates von Hippel-Lindau tumor-suppressor protein (VHL) Pro^402^ or Pro^564^, which allows HIF-1α to bind to the VHL. VHL interacts with the protein elongin C and subsequently ubiquitinates HIF-1α by recruiting an E3 ubiquitin-protein ligase complex. Thereafter, HIF-1α is targeted to the 26S proteasome for degradation [4]. HIF-1AN hydroxylates the Asn^803^ of HIF-1α and inhibits its interaction with the co-activator *p300*, which affects transcription of the downstream *VEGF* gene [4].

The reduced frequency of AMS in highlanders (HL) suggests that lowlanders (LL) are considerably more susceptible to AMS [5,6]. Genes that are associated with high altitude adaptation could influence AMS, as suggested by Wu *et al.* [6]. Evidence of genetic adaptation at different altitudes was recently provided by genome-wide investigations in HL and LL populations [7,8,9] and these investigations indicated that the *EGLN1* and *HIF-1AN* genes could be involved in AMS. Therefore, polymorphisms in these genes may explain inter-individual differences in AMS. Pharmaceuticals, such as dexamethasone [10] have been identified that prophylactically treat AMS symptoms. The identification of individuals who have a genetic predisposition for AMS would be beneficial for decision-making regarding prophylactic drug use.

The untranslated regions (UTRs) of eukaryotic genes contain regulatory regions. The 5' untranslated regions (5'-UTRs) influence the efficiency of translation and even have post-transcriptional effects, including effects on mRNA stability and folding [11]. Therefore, sequence variations in 5'-UTR introns may affect gene expression. SNPs located in the 3'-UTR may affect the binding affinities of miRNAs for their target mRNAs [12], thus affecting the expression and function of the target gene. In the present case-control study, we attempted to determine whether variants of the *EGLN1* and *HIF-1AN* genes, either previously identified in high altitude adaption study [13] or our currently selected in the UTRs, are associated with susceptibility to AMS.

Since AMS happens mostly in a population acutely exposed to hypoxic environment, we recruited male Han Chinese volunteers who had been living at low altitudes, without recent high altitude exposure history. These subjects took the flights from 500 m (Chengdu in Sichuan province) to 3700 m (Lhasa) within 2 h.

## 2. Results

### 2.1. Characteristics of the Study Population

A total of 380 DNA samples were genotyped, including 190 AMS patients and 190 controls. The characteristics of the study populations are shown in Table 1. No significant differences in age, height, weight, smoking rate or heart rate were noted between the AMS^+^ and AMS^−^ group. However, SaO_2_ significantly differed between the AMS^+^ and AMS^−^ groups (Table 1).

**Table 1:** Demographic characteristics and physiological parameters of study population.

Demographic Characteristics and Physiological Parameters	AMS^+^	AMS^−^	*p* Value
Age (year)	22.7 ± 3.5	22.9 ± 3.8	0.840
Height (cm)	171.7 ± 4.9	171.7 ± 4.8	0.940
Weight (kg)	64.7 ± 10.4	64.7 ± 10.4	0.830
Smoking rate (%)	49.5	53.1	0.472
Heart rate (bpm)	86.5 ± 12.9	84.7 ± 12.4	0.156
SaO_2_ (%)	88.1 ± 2.90	88.8 ± 2.98	0.013 *

bpm: beat per minute, * *p* < 0.05

### 2.2. Genotyping of Target Single Nucleotide Polymorphisms (SNPs)

The genotyping success rates of four target SNPs ranged from 98.7% to 100% in this study. Basic information regarding the SNPs in cases and controls is presented in Table 2. Of these SNPs, rs12757362, rs1339894 and rs1361384 exhibited very low minor allele frequencies (MAF) and were excluded from further analyses.

### 2.3. Genetic Models of the Egl Nine Homolog 1 (EGLN1) and Hypoxia-Inducible Factor 1-α Inhibitor (HIF-1AN) Genes and the Risk of Acute Mountain Sickness (AMS)

AMS is a complex disease and its mode of genetic inheritance is not clear. Codominant, dominant, recessive, overdominant and additive genetic models were applied to assess the association of SNPs within or near the *HIF-1AN* and *EGLN1* genes with the risk of AMS using SNPStats software. We found that rs12406290 and rs2153364 in the 5'-UTR of the *EGLN1* gene were associated with AMS risk (Table 3). The genetic models of the other polymorphisms are shown in Table 1 in the supplementary material.

**Table 2:** Basic single nucleotide polymorphism (SNP) information.

rs Number	Gene Name	Allele	Chromosome Position	MAF	HWE *p*
rs1054399	*HIF-1AN*	C/T	Chr10:100552808	0.185	0.187
rs11190613	*HIF-1AN*	C/T	Chr10:100554240	0.187	0.188
rs11292	*HIF-1AN*	C/T	Chr10:100553850	0.187	0.188
rs11816840	*HIF-1AN*	C/G	Chr10:100549463	0.182	0.188
rs3750633	*HIF-1AN*	A/G	Chr10:100548457	0.187	0.188
rs12406290	*EGLN1*	A/G	Chr 1:231423480	0.460	0.378
rs12757362	*EGLN1*	C/G	Chr 1:231426746	0.060	1.000
rs1339894	*EGLN1*	A/G	Chr 1:231424811	0.001	1.000
rs1361384	*EGLN1*	A/G	Chr 1:231424487	0.005	0.970
rs2009873	*EGLN1*	A/G	Chr 1:231363490	0.420	0.487
rs2153364	*EGLN1*	A/G	Chr 1:231424474	0.530	0.532
rs2486729	*EGLN1*	A/G	Chr 1:231399838	0.420	0.302
rs2739513	*EGLN1*	A/G	Chr 1:231379455	0.430	0.541

MAF: Minor allele frequency; HWE: Hardy-Weinberg equilibrium.

**Table 3:** Single nucleotide polymorphism genetic models and analyses of the association between SNPs and acute mountain sickness (AMS) adjusted for age and smoking status.

SNP Number	Model	Genotype	AMS^+^ [*n* (%)]	AMS^−^ [*n* (%)]	OR (95% CI)	*p*-Value	AIC	BIC
rs12406290	Codominant	AA	65 (34.6)	42 (22.7)	1.00	–	–	–
		AG	86 (45.7)	96 (51.9)	1.74 (1.07–2.84)	–	–	–
		GG	37 (19.7)	47 (25.4)	1.91 (1.06–3.41)	0.04	522.9	554.3
	Dominant	AA	65 (34.6)	42 (22.7)	1.00	–	–	–
		AG + GG	123 (65.4)	143 (77.3)	1.79 (1.13–2.84)	0.012 *	521	548.5
	Recessive	AA + AG	151 (80.3)	138 (74.6)	1.00	–	–	–
		GG	37 (19.7)	47 (25.4)	1.34 (0.82–2.19)	0.24	526	553.5
	Overdominant	AA + GG	102 (54.3)	89 (48.1)	1.00	–	–	–
		AG	86 (45.7)	96 (51.9)	1.31 (0.87–1.97)	0.19	525.7	553.1
	Log-additive	–	–	–	1.40 (1.05–1.87)	0.022	522.1	549.6
rs2153364	Codominant	AA	63 (33.5)	38 (21.1)	1.00	–	–	–
		AG	88 (46.8)	95 (52.8)	1.85 (1.12–3.05)	–	–	–
		GG	37 (19.7)	47 (26.1)	2.14 (1.18–3.88)	0.019	514.4	545.6
	Dominant	AA	63 (33.5)	38 (21.1)	1.00	–	–	–
		AG + GG	125 (66.5)	142 (78.9)	1.94 (1.20–3.11)	0.0057 *	512.7	540
	Recessive	AA + AG	151 (80.3)	133 (73.9)	1.00	–	–	–
		GG	37 (19.7)	47 (26.1)	1.43 (0.88–2.34)	0.15	518.2	545.6
	Overdominant	AA + GG	100 (53.2)	85 (47.2)	1.00	–	–	–
		AG	88 (46.8)	95 (52.8)	1.30 (0.86–1.96)	0.22	518.8	546.1
	Log-additive	–	–	–	1.47 (1.10–1.98)	0.0095	513.6	540.9

Numbers and frequencies of AMS^+^ or AMS^−^ in each genotype were shown. AIC: Akaike’s information criterion; BIC: Bayesian information criterion. * Best-fit model *p*-value.

Under the codominant model, the genotypes “AG” (OR = 1.74; 95% CI, 1.07–2.84; *p* = 0.04) and “GG” (OR = 1.91; 95% CI, 1.06–3.41; *p* = 0.014) of rs12406290 were associated with an increased AMS risk. Under the dominant model, the “AG + GG” genotype of rs12406290 was associated with an increased AMS risk (OR = 1.79; 95% CI, 1.13–2.84; *p* = 0.012). In the log-additive model, rs12406290 was associated with an increased risk of AMS (OR = 1.40; 95% CI, 1.05–1.87; *p* = 0.022) (Table 3).

Similarly, under the codominant model, the “AG” (OR = 1.85; 95% CI, 1.12–3.05; *p* = 0.019) and “GG” (OR = 2.14; 95% CI, 1.18–3.88; *p* = 0.019) genotypes of rs2153364 were associated with an increased AMS risk compared with the “AA” genotype. Under the dominant model, the “AG + GG” genotype of rs2153364 was associated with an increased AMS risk (OR = 1.94; 95% CI, 1.20–3.11; *p* = 0.0057). Under the log-additive model, rs2153364 was associated with an increased risk of AMS (OR = 1.47; 95% CI, 1.10–1.98; *p* = 0.0095) (Table 3).

The model with the lowest AIC and BIC values for a given polymorphism was considered the best-fit model. The AIC and BIC values indicated that the dominant model may serve as the best-fit model of rs12406290 and rs2153364 (Table 3). However, these values were not significant after Bonferroni correction (*p*_correction_ = 0.05/13 = 0.0038).

### 2.4. HIF-1AN and EGLN1 Haplotypes and the Risk of AMS

**Figure 1:**
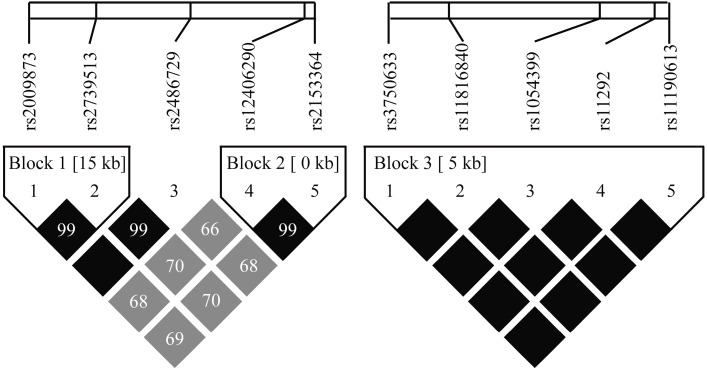
Linkage disequilibrium (LD) plot constructed by Haploview 4.2 software.

The linkage disequilibrium (LD) coefficients were calculated using the Gabriel algorithm [14]. The results indicated that there were two LD blocks in the *EGLN1* gene and one LD block in the *HIF-1AN* gene. Block 1 consisted of rs2009873-rs2739513 (Figure 1). Two common haplotypes (frequencies of ≥0.05 for each) with a cumulative frequency of 0.991 were identified. No significant difference was observed between the two main haplotypes (Table 4). Block 2 consisted of rs12406290-rs2153364, which is located upstream of the *EGLN1* gene (Figure 1). Two common haplotypes (frequencies of ≥0.05 for each) with a cumulative frequency of 0.993 were identified in block 2. Carriers of the “GG” haplotype exhibited an increased risk of AMS after adjusting for age and smoking status (OR = 1.38; 95% CI, 1.04–1.85; *p* = 0.029) (Table 4). Block 3 consisted of rs1054399, rs11190613, rs11292, rs11816840 and rs3750633 in the 3'-UTR region of the *HIF-1AN* gene (Figure 1). Two common haplotypes (frequencies ≥0.05 for each) with a cumulative frequency of 1 were identified. No significant difference was observed between the two haplotypes after adjusting for age and smoking status (Table 4).

The LD plot was generated using genotyping data from this study and Haploview 4.2 software. The correlation between two SNPs is indicated by the gray scale (a darker color indicates a strong correlation) by pairwise *r*^2^ values, and the pairwise *D*’ values are given as the percentage in each cell (cell in pure black indicates *D*’ = 1.00). Block 1 was formed by rs2009873-rs2739513. Block 2 was formed by rs12406290-rs2153364, which are located upstream of the *EGLN1* gene. Block 3 consists of rs1054399, rs11190613, rs11292, rs11816840 and rs3750633, which are located in the 3'-UTR of the *HIF-1AN* gene.

## 3. Discussion

In the present study, 13 SNPs in two low-oxygen sensor genes that regulate HIF-1α were genotyped. Our study determined that rs12406290 and rs2153364 in the 5'-UTR of the *EGLN1* gene are strongly associated with AMS risk after adjusting for age and smoking status, even though after Bonferroni correction the results turned out to be insignificant. AIC and BIC analyses indicated that a dominant model may serve as the best-fit genetic model for these SNPs. Further haplotype analyses found that the rs12406290-rs2153364 haplotype was associated with AMS. The results of the current study provide novel information about *EGLN1* under hypobaric hypoxia that might reveal a role for this gene in AMS. No significant association was found between polymorphisms in the *HIF-1AN* 3'-UTR and AMS before and after Bonferroni correction. The prevalence of risk genotypes and haplotypes in AMS supports the hypothesis regarding the involvement of common genetic factors.

**Table 4:** Haplotype frequencies and the association with AMS risk adjusted for age and smoking status.

Gene	Blocks	Haplotype ^a^	Frequency of AMS^−^ (%)	Frequency of AMS^+^ (%)	*p-*Value	Odds Ratio 95% CI
***EGLN1***	Global haplotype association test *p* = 0.004 ^c^					
	Block 1	AA	60.9	54.75	–	1.00 ^b^
		GG	39.1	43.63	0.12	1.25 (0.94–1.66)
	Global haplotype association test *p* = 0.036 ^c^					
	Block 2	AA	56.83	48.92	–	1.00 ^b^
		GG	42.1	50.79	0.029 *	1.38 (1.04–1.85)
***HIF-1AN***	Global haplotype association test *p* = 0.7 ^c^					
	Block 3	CTTGG	90.26	90.63	–	1.00 ^b^
		TCACA	9.74	9.37	0.7	0.91 (0.57–1.46)

Frequency of each haplotype block was shown in percentage. ^a^, Haplotypes with frequency ≥0.05 were shown; ^b^, Reference haplotype; ^c^, A global test was performed for the null hypothesis such that no haplotype was associated with the risk of AMS. The analyses were adjusted by age and smoking status. * *p* < 0.05; Block 1 consisted of rs2009873-rs2739513; Block 2 consisted of rs12406290-rs2153364; Block 3 consisted of rs1054399, rs11190613, rs11292, rs11816840 and rs3750633.

At higher altitudes, the atmospheric mass at the measurement point indexed to sea level is reduced, which results in decreased barometric pressure. Here, a decline in the partial pressure of oxygen was observed. AMS commonly develops in individuals who arrive at altitudes greater than 2500 m above sea level and is accompanied by a decrease in SaO_2_ [15]. Our results also indicate that AMS patients have a lower SaO_2_ at plateau. HIF-1α subunits have a short half-life under normoxic conditions, as HIF-1α is synthesized and degraded continuously in cells. However, the degradation of *HIF-1α* is reduced under hypoxia [16]. HIF-1α accumulation and subsequent up-regulation of VEGF could contribute to basement membrane damage, and exaggerated edema is involved in the pathophysiological process, as suggested by Wilson *et al.* [3]. A recent study indicates that dexamethasone impairs HIF-1α function and the expression of its target genes [17]. This finding may explain the fact that the prophylactic use of dexamethasone efficiently reduces the occurrence of AMS.

Studies have revealed that EGLN1 activity is reduced under hypoxic conditions by substrate limitation [18] or catalytic center inhibition [19]; thus, EGLN1 plays a key role in HIF-1α regulation and downstream gene expression. Numerous studies have revealed that *EGLN1* gene expression is associated with high altitude adaption in various ethnicities at different plateaus [7,8,13,20,21]. An *in vitro* study demonstrated that the *PHD2* haplotype D4E/C127S dramatically diminished the interaction between PHD2 and p23, leading to impaired PHD2-mediated down-regulation of the HIF pathway [22]. In addition, the Asp4Glu; Cys127Ser variant promotes increased HIF-1 degradation under hypoxic conditions [23]. A case-control study with a relatively small sample size revealed that two common *EGLN1* variants are associated with AMS. Our present study suggests that the rs12406290 and rs2153364 SNPs located in the 5'-UTR of *EGLN1* are associated with AMS in a Han Chinese population. At a type I error probability of 0.05, the statistical power to detect a relative risk of 2 compared with the AMS^+^ group was sufficient for rs12406290 and rs2153364. Thus, variation in the 5'-UTR may affect the level of gene expression and protein translation. The online software programs FuncPred (http://snpinfo.niehs.nih.gov/) and F-SNP (http://compbio.cs.queensu.ca/F-SNP/) were used to predict the functional effects of these two SNPs. The functional prediction results indicated that rs12406290 and rs2153364 are both located at the presumed transcription factor binding site of the *EGLN1* gene. We further used TFSEARCH Search software (http://www.cbrc.jp/research/db/TFSEARCH.html) to identify TFs. Our analysis revealed that the rs12406290 and rs2153364 polymorphisms were located at binding sites for HSG and c-Ets, respectively. Thus, rs12567209, rs2153364 and rs12406290-rs2153364 haplotypes may alter transcription factor binding sites and affect *EGLN1* gene expression to influence HIF-1α regulation.

HIF-1AN hydroxylates HIF-1α and inhibits its interaction with p300, thus regulating the HIF pathway. Loss of HIF-1AN significantly alters the physiological response and causes a hyper-metabolic phenotype [24]. Additionally, the response of the brain to altitude is crucial due to its high rate of oxygen consumption. A decrease in the partial pressure of inhaled oxygen at high altitudes in combination with a hyper-metabolic phenotype might induce AMS. HIF-1AN is associated with advanced prostate cancer due to its regulatory effects on HIF-1 [25]. miRNAs that target *FIH-1* have been shown to regulate metabolism [26]. We therefore genotyped 3'-UTR variants to determine their relationship to AMS. Further analyses with the online software program MirSNP (http://cmbi.bjmu.edu.cn/mirsnp) indicate that rs11292 and rs11816840 are located at a predicted miRNA binding site. However, no associations were identified between AMS and rs1054399, rs11190613, rs11292, rs11816840 or rs3750633.

It is possible that the results of the current study may only be applied to young males because the study subjects were all young males. Our study is the first to demonstrate that the rs12406290, rs2153364 and rs12406290-rs2153364 haplotypes in the 5'-UTR of the *EGLN1* gene are associated with AMS risk in a Han Chinese population. These results further demonstrate that genes associated with high altitude adaptation may also be involved in AMS. Further functional characterization of the effects of the polymorphisms on transcription factor binding and EGLN1 expression may elucidate the underlying mechanisms involved in AMS etiology. Prophylactic drug use may be beneficial for individuals with “G” alleles of rs12406290 and rs2153364 and a “GG” haplotype before they ascend to high altitudes.

## 4. Methods

### 4.1. Subjects

The subjects included 190 patients with AMS and 190 healthy Han Chinese controls randomly chosen from 850 volunteers who ascended from 500 m (Chengdu in Sichuan province) to 3700 m (Lhasa) in 2 h by plane. All included subjects were unrelated males who had lived at low altitudes prior to being recruited during the period from 21 June to 25 June 2012, as previously described [27]. Subjects who were exposed to altitudes ≥2500 m in the past 6 months, subjects who were taking acetazolamide or steroids for AMS prophylaxis and subjects who were diagnosed with psychological or neurological disorders were excluded. Venous blood (5 mL) was obtained from all individuals included in the study for future genetic analysis, and written informed consent was obtained from all subjects. The study was approved by the Ethics Committee of Xinqiao Hospital, Third Military Medical University (identification code: 2012014 approved on 9 May 2012).

### 4.2. AMS Score and Physical Signs

AMS scores were obtained using the Lake Louise scoring system (LLss) within 24 h after the participants arrived at 3700 m. The subjects were then divided into AMS^+^ and AMS^−^ individuals in accordance with the LLSS results. Smoking status was classified as follows: 0 = no smoking history, 1 = past smoking history and 2 = present smoking. Physiological signs of HR and BP were measured using a sphygmomanometer (HEM-6200, OMRON Healthcare Ltd., Kyoto, Japan). SaO_2_ was measured using a Pulse Oximeter (NONIN-9550, Nonin Onyx, Plymouth, MN, USA).

### 4.3. SNP Selection

We performed a search on website (http://www.ncbi.nlm.nih.gov/projects/SNP/) and found that *HIF-1AN* exhibits a wide range of 3'-UTR sequences. We selected rs1054399, rs11190613, rs11292, rs11816840 and rs3750633 SNPs, which located in the *HIF-1AN* 3'-UTR with Minor Allele Frequency (MAF) ≥0.1. Similarly, we selected rs12406290, rs12757362, rs1339894, rs2153364 and rs1361384 SNPs at *EGLN1* 5'-UTR, and rs2009873 near *EGLN1* 3'-UTR, based on MAF values ≥0.1. A genome-wide association study for high altitude adaption [13] indicates that six *EGLN1* intron SNPs (rs2066140, rs2739513, rs2486736, rs480902, rs2790882 and rs2486729) are listed in the top 0.01% of highly differentiated SNPs between Tibetans and Han Chinese. Because they are all in linkage disequilibrium (LD) (threshold r^2^ ≥ 0.8) in CHB by tagger-pairwise algorithm in Hapmap (http://hapmap.ncbi.nlm.nih.gov/), we selected two SNPs (rs2739513 and rs2486729) as tag SNPs.

### 4.4. Genotyping

Genomic DNA was extracted from whole blood in accordance with the instructions provided with the PAXgene Blood DNA kit (Qiagen, Hilden, Germany). Genomic DNA samples were stored at −20 °C until analysis. PCR primer pairs were designed using Sequenom^®^ Assay Design software (version 3.1, Sequenom Inc., San Diego, CA, USA) and synthesized by Sangon Biotech (Shanghai, China; see Table S1). Genotyping was performed using the MassARRAY^®^ MALDI-TOF System (Sequenom Inc., San Diego, CA, USA). For quality control, two non-template controls were used in the plate and chip.

### 4.5. Data Analysis

SHEsis (http://analysis.biox.cn/myAnalysis.php) online software was used to confirm Hardy–Weinberg equilibrium and allele frequencies via Chi-square tests. Differences in the physiological parameters of the study population were assessed by *t*-tests as appropriate. Analyses of genotype distributions adjusted for age and smoking status were performed by unconditional logistic regression. AIC and BIC values were used to determine the best-fit model for each SNP. LD analysis and haplotype construction using Haploview 4.2 [28] software were subsequently employed to further analyze the association of different haplotypes with the risk of AMS. The results were analyzed using SPSS 16.0 (SPSS Inc., Chicago, IL, USA) and SNPStats online software (http://bioinfo.iconcologia.net/SNPstats) [29]. Power calculations were performed using power and sample size calculations [30].

## 5. Conclusions

Our study indicates that variants in the *EGLN1* 5'-UTR influence the susceptibility to AMS in a Han Chinese population.

## Supplementary Materials

Supplementary materials can be found at http://www.mdpi.com/1422-0067/15/12/21777/s1.
